# Identification of potentially inappropriate medications characteristic of older individuals with diabetes: a study using pharmacy claims data

**DOI:** 10.1186/s40780-025-00505-7

**Published:** 2025-11-12

**Authors:** Takashi Yamamoto, Yoshihito Kasanami, Tomoyoshi Miyamoto, Sumio Matzno, Mikio Sakakibara, Atsufumi Kawabata

**Affiliations:** 1 Faculty of Pharmacy, Kindai University, 3-4-1 Kowakae, Higashi-Osaka, 577-8502 Japan; 2 Sugi Pharmacy Co., Ltd., 62-1 Shinye, Yokone-cho, Obu, 474-0011 Japan; 3 School of Pharmacy, Hyogo Medical University, 1-3-6 Minatojima, Chuo-ku, Kobe, 650-8530 Japan

**Keywords:** Polypharmacy, Potentially inappropriate medications, Diabetes, Elderly, Geriatrics

## Abstract

**Background:**

Older patients with diabetes often require multiple medications to manage complications, and have a high risk of potentially inappropriate medications (PIMs). Given limited information regarding diabetes-specific PIMs, we characterized PIM prescriptions in older patients with and without type 1 or 2 diabetes, and examined gender differences in diabetes-specific PIMs, using pharmacy claims data obtained from nationwide branches of a chain pharmacy company in Japan.

**Methods:**

Pharmacy claims data of patients aged 65 years and over who used one of the 905 community-based pharmacy branches of Sugi Pharmacy Co., Ltd. during a month were anonymized and analyzed. The enrolled individuals were separated to diabetic and non-diabetic groups on the basis of anti-diabetic medications. The two groups were balanced for the age, gender ratio, and status of home healthcare insurance application through propensity score matching (PSM). PIMs, i.e. medications prone to cause adverse reactions and replaceable with others, were classified to twelve categories other than anti-diabetic PIMs, and the incidence of each PIM category in diabetic and non-diabetic groups was analyzed.

**Results:**

Of 333,869 older patients, 37,606 received anti-diabetic medications. Analysis of pharmacy claims data after PSM showed that aged individuals with diabetes had significant increases in the number of medications and the incidence of PIMs, compared to individuals without diabetes. Multivariate logistic regression analyses indicated that older individuals with diabetes had significantly increased incidences of six PIM categories, sleeping drugs, α-adrenoceptor blockers, H_2_-blockers, drugs for overactive bladder, diuretic drugs, and concomitant intake of multiple antithrombotic drugs. Sub-group analyses of each gender data detected a diabetes-related significant increase in the incidence of the PIM category of drugs for overactive drugs in older females, but not males [adjusted odds ratios (95% confidence interval): 1.59 (1.35–1.88) and 0.91 (0.77–1.08), respectively], while significantly increased incidences of the residual five PIM categories in the diabetic group were detected regardless gender.

**Conclusion:**

Older individuals with diabetes had higher risks of five PIM categories regardless of gender, and of one female-specific PIM category, than individuals without diabetes. These findings are helpful for pharmacists to make recommendations of deprescribing PIMs in pharmaceutical care of older patients with diabetes Geriatrics.

**Supplementary Information:**

The online version contains supplementary material available at 10.1186/s40780-025-00505-7.

## Background

Diabetes remains a substantial public health issue worldwide. In particular, type 2 diabetes prevalence is greatly increasing worldwide, primarily due to the increased obesity, and pharmacological approaches to preventing and controlling type 2 diabetes are essential to reduce the incidence or exacerbation of diabetic complications. Complex medication management challenges to treat diabetic complications might increase the risk of polypharmacy and potentially inappropriate medications (PIMs) [1]. In particular, older patients with type 2 diabetes are prone to polypharmacy, and have a greater risk of receiving PIMs [2].

The American Geriatric Society (AGS) Beers Criteria [3] and STOPP (Screening Tool of Older Person’s Prescriptions) / START (Screening Tool to Alert doctors to Right Treatment) [4] have been developed as screening tools for PIM. The Japanese Geriatrics Society has published a list of PIMs as the “Screening Tool for Older Person’s Appropriate Prescriptions for Japanese”, which is useful to improve the provision of medical care for older adults in Japan [5, 6]. In general, pharmacists are expected to detect the use of PIMs and make an appropriate deprescribing recommendation in pharmacotherapy for older adult patients [7–9]. Most recently, we identified PIMs characteristic of older patients with dementia, which were greatly different from PIMs used for those without dementia [10]. Thus, specific disease conditions might characterize the pattern of PIM prescriptions in relation to their complications, altered drug metabolism, medication contraindications, and complex drug interactions. Particularly, diabetes would induce a specific pattern of PIM prescriptions because of common multimorbidities with diabetes, such as renal and retinal impairment, peripheral neuropathy, and cardiovascular diseases. Thus, it is rationale to characterize diabetes-specific PIMs and develop the criteria of recommendations for deprescribing of PIMs to assist pharmacist-led optimization of pharmacotherapy. Although several studies have reported PIMs in individuals with diabetes [11, 12], diabetes-specific PIMs have not been clearly characterized [13]. Furthermore, sex differences in the pathophysiology of diabetes including the severity of diabetic complications and the treatment efficacy or side effects [14–16] remind us that older males and females with diabetes might have different patterns of risks in PIM prescriptions. Since currently available PIM guidelines lack discussion of sex-related differences [17–19], characterization of sex disparities in diabetes-specific PIMs is an urgent need. In the present study, we thus focused on characterization of PIMs that need special attention for older patients with diabetes, and analyzed pharmacy claims data of nationwide 905 community-based pharmacies of Sugi Pharmacy Co., Ltd.

## Methods

### Collection of pharmacy claims data and the PIMs list employed

We conducted a retrospective cross-sectional study using pharmacy claims data collected from nationwide 905 community-based branches of Sugi Pharmacy Co., Ltd. (Obu, Aichi, Japan), all of which regularly accepted prescriptions from any type of medical institutions providing primary, secondary or tertiary care. The pharmacy claims data included prescription-related information, such as prescribed drugs, patients’ age and sex, and medical fees, of patients aged 65 years and over, who visited one of the pharmacy branches during one month starting from December 1st, 2019. This data collection period was just due to the data availability but not special reason. To ensure anonymity, all prescription records were coded by an outsider, prior to analysis, and managed by a unique number allotted to each participant. There was no duplicate sampling, because each drug was counted only once per patient per month, regardless of prescription frequency or changes. For patients who had multiple visits or prescription modifications during the month, each unique drug was recorded as a single prescribed medication for each individual. We used a list of PIMs prepared for older adult patients in Japan [5, 10], but did not analyze PIMs used for treatment of diabetes itself, which are never prescribed for individuals without diabetes (Supplementary materials; Table S1).

### Classification of diabetic and non-diabetic groups and propensity score matching

Patients were divided into two groups, i.e., older individuals with and without type 1 or 2 diabetes, by checking prescribed anti-diabetic medications (Supplementary materials; Table S2), because pharmacy claims data available in Japan do not include information about diagnoses for each patient. A group of “individuals with type 1 or 2 diabetes” was defined as patients receiving any medication used for treatment of type 1 or 2 diabetes, as follows: insulin, glucagon-like peptide-1 receptor agonists, sulfonylureas, glinides, biguanides, alpha-glucosidase inhibitors, DPP-4 inhibitors, sodium-glucose cotransporter-2 inhibitors, insulin sensitizers, and anti-diabetic drug combinations (Supplementary Table S2). The other group, “individuals without type 1 or 2 diabetes”, was defined as patients who did not receive any of the above-mentioned medications for type 1 or 2 diabetes. It is to be noted that patients who were diagnosed with diabetes but did not receive diabetes medications would be designated into the non-diabetic group, which was one of limitations in the present study using Japanese pharmacy claims data. To reduce biases, propensity score matching (PSM) was performed to match patients with comparable baseline characteristics between diabetic and non-diabetic groups. The covariates introduced into our model were age, sex and status of application of home healthcare-related insurance (i.e., additional insurance coverage according to “Guidance for the management of in-home medical long-term care” or “Home care patient drug management guidance”), and the caliper coefficient was set to 0.2. PSM between diabetic and non-diabetic groups was also conducted before sub-group analysis in each of genders. It is to be noted that “age”, “sex”, and “home healthcare insurance” are only available information other than prescriptions in Japanese pharmacy claims data, while information of common chronic conditions that should be ideally included as confounding factors in matching is not available in the pharmacy claims data. Nevertheless, “age”, “sex”, and “home healthcare insurance” are closely related with the development and prognosis of diabetes [20–24], and considered useful for balancing diabetic and non-diabetic groups.

### Statistical analysis for identification of PIMs characteristic of diabetes in older patients

Before PSM, patients’ characteristics, age, gender ratio and status of home healthcare insurance application, between diabetic and non-diabetic groups, were statistically compared using Mann-Whitney *U* test or Fisher’s exact test. After PSM, difference of the number of prescribed medicines other than anti-diabetic agents between the two groups was evaluated by Mann-Whitney *U* test. To analyze the differences of incidence of PIMs between diabetic and non-diabetic older patients, we focused on PIMs other than anti-diabetic drugs, which were designated to all aged individuals, but not to ones with special diseases or conditions. The PIM categories analyzed in this study were: “sleeping drugs”, “NSAIDs”, “α-blockers”, “anti-Parkinson drugs”, “1st generation H_1_-blockers”, “H_2_-blockers”, “drugs for overactive bladder”, “anti-depressants”, “anti-emetics”, “diuretic drugs”, “sulpiride” and “concomitant intake of multiple antithrombotic drugs” (Supplementary materials; Table S1). The incidence of each of the PIM categories was statistically compared between diabetic and non-diabetic groups by univariate and multivariate logistic regression analyses. Similar sub-group analysis in each gender was performed after PSM. The collinearity was examined using the variance inflation factor (VIF). The variable we used for all multivariate analyses was VIF < 5. Odds ratios (ORs) are shown with 95% confidence intervals (CI). Statistical significance was set at *p* value < 0.05. Statistical analyses were performed using EZR (Saitama Medical Center, Jichi Medical University, Saitama, Japan) [25], which is a graphical user interface for R (The R Foundation for Statistical Computing, Vienna, Austria version 4.1.2). More precisely, it is a modified version of R commander (version 2.7-1) designed to easily execute statistical functions frequently used in biostatistics. Post-hoc power analysis revealed that our propensity score-matched sample (37,606 patients per group) provided >90% power to detect the observed effect sizes for major PIM categories (α = 0.05, two-tailed test), confirming adequate statistical power for our primary analyses.

### Ethical consideration

The protocol of this study was in accordance with the ethical principles of the Declaration of Helsinki, and was approved by the Ethics Committees of the Faculty of Pharmacy, Kindai University (approval number 20–161, April 22nd, 2020), which waived the requirement for informed consent owing to the retrospective nature of our study and gave approval for the use of an opt-out strategy concerning patient consent.

## Results

### Characterization of patients enrolled in this study and propensity score-matching

Of 333,869 patients aged 65 years and older who visited 905 community-based pharmacies of Sugi Pharmacy Co., Ltd. during 1 month of December 31, 2019, 37,606 (11.2%) undergoing anti-diabetic pharmacotherapy were defined as individuals with diabetes and categorized to a diabetic group, while residual 296,263 (88.7%) patients were designated to a non-diabetic group (Table 1). The age and gender ratio (female/male) were significantly lower in the diabetic group than the non-diabetic group, although there was no difference in the status of home healthcare insurance application between the two groups (Table 1). Using PSM, the baseline covariates including the age, gender ratio and status of application of home healthcare insurance, were balanced between diabetic and non-diabetic groups, and thereafter the data of 37,606 patients in each group were subjected to the following statistical analysis.

**Table 1:** Patient characteristics before and after propensity score matching

Characteristics		Before propensity score matching			After propensity score matching		
		Diabetes			Diabetes		
		(-)	(+)		(-)	(+)	
		*n* = 296,263	*n* = 37,606	*p*	*n* = 37,606	*n* = 37,606	*p*
Age, Median [range]		76 [65–107]	74 [65–107]	**< 0.001**	74 [65–103]	74 [65–107]	1.00
Gender, n (%)	Female	171,911 (58.0)	16,396 (43.6)	**< 0.001**	16,398 (43.6)	16,396 (43.6)	0.99
	Male	124,352 (42.0)	21,210 (56.4)		21,208 (56.4)	21,210 (56.4)	
Use of home healthcare insurance, n (%)	No	279,032 (94.2)	35,465 (94.3)	0.343	35,465 (94.3)	35,465 (94.3)	1.00
	Yes	17,231 (5.8)	2141 (5.7)		2141 (5.7)	2141 (5.7)	

Statistical comparisons of age between diabetes and non-diabetes groups were performed using Mann-Whitney’s *U* test, and the differences in the gender ratio and status of home healthcare insurance application between the two groups were analyzed by Fisher’s exact test

### Polypharmacy and increased incidence of PIMs in older adults with diabetes

**Fig. 1:**
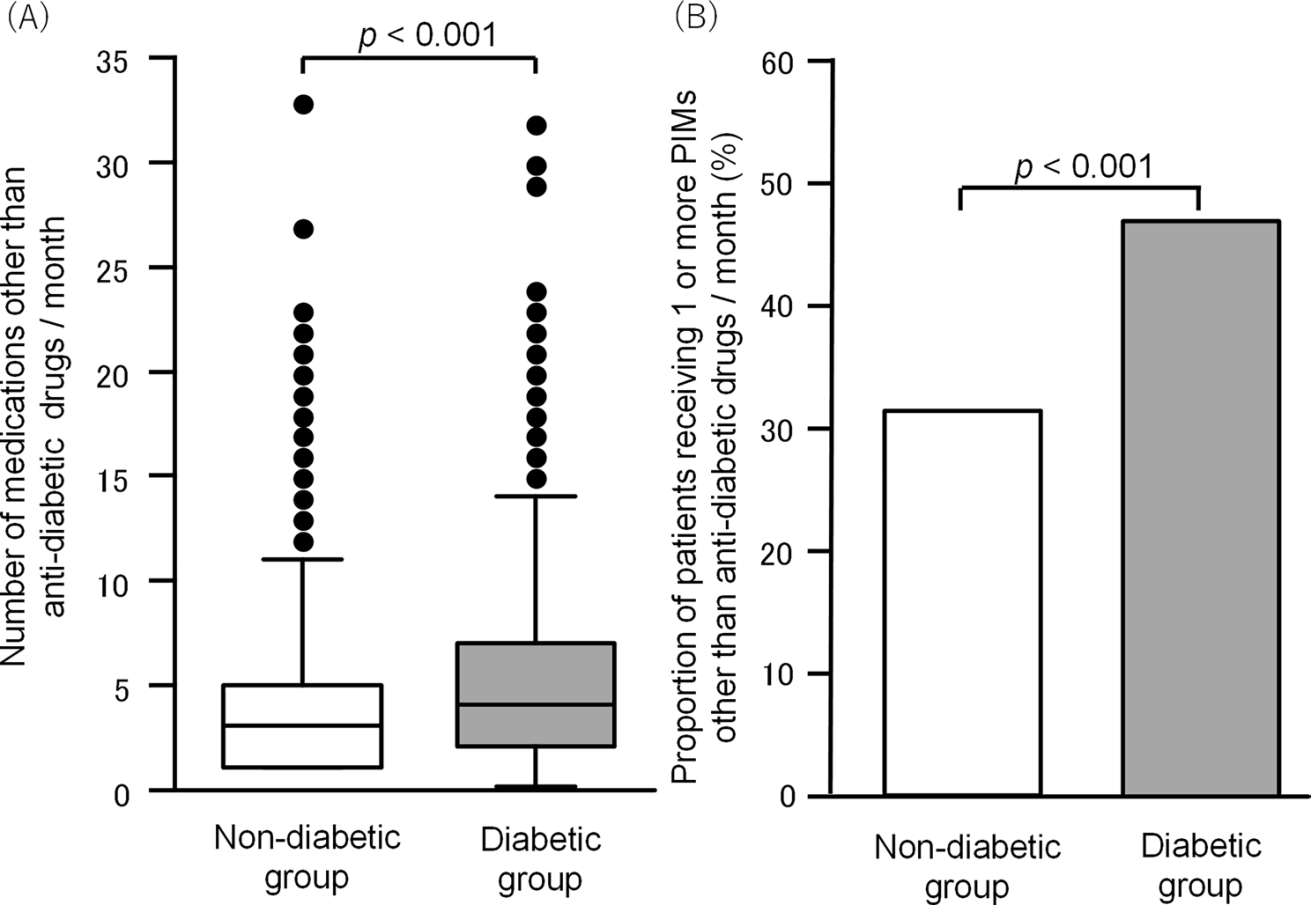
Polypharmacy and increased incidence of PIMs in older patients with diabetes. (**A**) The number of medications other than anti-diabetic drugs prescribed over a period of 1 month in diabetic and non-diabetic groups after propensity score matching. Data show the median with upper and lower quartiles, as well as the maximum and minimum values, while outliers are indicated as dots. (**B**) Proportion of patients receiving PIMs other than anti-diabetic drugs over a period of 1 month in diabetic and non-diabetic groups. Statistical analysis of the differences between diabetic and non-diabetic groups was performed using Mann-Whitney’s *U* test (**A**) or Fisher’s exact test (**B**)

To ascertain the previous evidence that diabetes is one of major causes of polypharmacy, which is generally defined as the concurrent use of 6 or more medications, especially in older adults [2, 26, 27], the number of prescribed medications other than anti-diabetic agents was compared between diabetic and individuals without diabetes after PSM. The median number of medications, except for anti-diabetic drugs, was 4 and 3 in the diabetic and non-diabetic groups, respectively, and there was a significant difference between the two groups (Fig. 1A). The polypharmacy in older adults with diabetes is understandable and considered unavoidable, because of so-called diabetes-related complications that may require additional medications. The incidence of PIMs other than anti-diabetic drugs in older adults with diabetes was also significantly higher than that in those without diabetes (Fig. 1B). Considering potential overestimation of the number of medications in some patients who visited the same pharmacy twice or more, we also conducted the same analysis using the data of prescriptions only in the first visit of each patient during the one-month observation period, and found that the results (Supplementary materials; Fig. S1) were almost the same as those in Fig. 1.

### Characterization of PIM categories prescribed in older adults with diabetes

We used univariate and multivariate logistic regression analyses to identify PIM categories that were more frequently prescribed in older adults with diabetes than those without diabetes. Of 12 categories of PIMs, 10 exhibited significantly different frequency between diabetic and individuals without diabetes (Table 2). Older adults with diabetes had increased incidence of six PIM categories, i.e. sleeping drugs, α-adrenoceptor blockers, H_2_-blockers, drugs for overactive bladder, diuretic drugs, and concomitant intake of multiple antithrombotic drugs, showing adjusted odds ratios (aORs), 1.22 (95% CI, 1.17–1.28), 1.96 (1.73–2.21), 1.47 (1.37–1.59), 1.22 (1.08–1.37), 1.72 (2.16–2.30), and 2.23 (2.16–2.30), respectively. In contrast, the incidence of four PIM categories, i.e. NSAIDs, anti-Parkinson drugs, anti-emetics, and sulpiride, was significantly lower in older adults with diabetes than those without diabetes, showing aORs of 0.56 (0.53–0.60), 0.59 (0.41–0.86), 0.41 (0.31–0.54), and 0.57 (0.45–0.73), respectively.

**Table 2:** Comparisons of prescription rates of each of PIMs categories after propensity score matching between diabetic and non-diabetic elderly patients

Category	Diabetes		Univariate analyses		Multivariate analysis	
	(-)	(+)				
	*n* = 37,606 (%)	*n* = 37,606 (%)	Odds ratio (95% Cl)	*p*	Adjusted odds ratio (95% Cl)	*p*
Sleeping drugs, n (%)	4024 (10.7)	5053 (13.4)	1.3 (1.24–1.35)	**< 0.001**	1.22 (1.17–1.28)	**< 0.001**
NSAIDs, n (%)	2516 (6.7)	1418 (3.8)	0.55 (0.51–0.58)	**< 0.001**	0.56 (0.53–0.60)	**< 0.001**
α-blockers, n (%)	388 (1.0)	925 (2.5)	2.42 (2.15–2.73)	**< 0.001**	1.96 (1.73–2.21)	**< 0.001**
Anti-Parkinson drugs, n (%)	90(0.2)	45(0.1)	0.50 (0.35–0.71)	**< 0.001**	0.59 (0.41–0.86)	**< 0.001**
1st generation H_1_-blockers, n (%)	228(0.6)	209 (0.2)	0.92 (0.76–1.11)	0.36	0.90 (0.73–1.09)	0.27
H_2_-blockers, n (%)	1216(3.2)	2096 (5.6)	1.77 (1.64–1.90)	**< 0.001**	1.47 (1.37–1.59)	**< 0.001**
Drugs for overactive bladder, n (%)	519 (1.4)	678(1.8)	1.31 (1.17-0.1.47)	**< 0.001**	1.22 (1.08–1.37)	**< 0.001**
Anti-depressants, n (%)	88(0.2)	65 (0.2)	0.74 (0.54–1.02)	0.06	0.74 (0.53–1.04)	0.08
Anti-emetics, n (%)	200(0.5)	72 (0.2)	0.36 (0.27–0.47)	**< 0.001**	0.41 (0.31–0.54)	**< 0.001**
Diuretic drugs, n (%)	1828 (4.9)	4339 (11.5)	2.55 (2.41–2.70)	**< 0.001**	1.72 (1.62–1.83)	**< 0.001**
Sulpiride, n (%)	188 (0.5)	111 (0.3)	0.59 (0.47–0.75)	**< 0.001**	0.57 (0.45–0.73)	**< 0.001**
Concomitant intake of multiple antithrombotic drugs, n (%)	763(2.0)	2373 (6.3)	2.47 (2.39–2.55)	**< 0.001**	2.23 (2.16–2.30)	**< 0.001**

Proportions of patients who received each of PIMs categories in diabetic and non-diabetic groups were analyzed using univariate and multivariate logistic regression analyses. CI, confidence interval

### Sub-group analyses to identify gender-specific PIM categories that need special attention in older adults with diabetes

To identify gender-specific increase in the incidence of PIM categories associated with diabetes in older adults, we performed sub-group analyses in each of gender. Before PSM, the diabetic group was slightly younger than the non-diabetic group in older females (Supplementary materials; Table S3) and in older males (Supplementary materials; Table S4). The proportion of older individuals with diabetes who applied for home healthcare insurance was significantly greater than those without diabetes in females (Supplementary materials; Table S3), but not males (Supplementary materials; Table S4). The baseline covariates, i.e. age and status of home healthcare insurance application, were balanced by PSM between diabetic and non-diabetic groups in each gender, and the data of 16,396 women with diabetes and 16,396 women without diabetes, and of 21,210 men with diabetes and 21,210 men without diabetes (Supplementary materials; Tables S3 and S4, respectively) were separately subjected to the sub-group analyses. The sub-group analyses in older females using univariate and multivariate logistic regression models detected significant increases in the incidence of six PIM categories in older females with diabetes (Table 3), which were identical to those detected by the analysis in both males and females (see Table 2). On the other hand, the sub-group analyses by univariate and multivariate logistic regression models in older males detected significant increases in the incidence of five PIM categories, i.e., sleeping drugs, α-adrenoceptor blockers, H_2_-blockers, diuretic drugs, and concomitant intake of multiple antithrombotic drugs, but did not show any increase in the incidence of a PIM category, drugs for overactive bladder (Table 4). The aORs for the increased incidence of drugs for overactive bladder in the patients with diabetes, as calculated separately in each gender by multivariate analyses, were 1.59 (1.35–1.88) in older females (Table 3), and 0.91 (0.77–1.08) in older males (Table 4).

**Table 3:** Sub-group analysis in females: comparisons of prescription rates of each of PIMs categories after propensity score matching between diabetic and non-diabetic elderly women

Category	Diabetes		Univariate analyses		Multivariate analysis	
	(-)	(+)				
	*n* = 16,396 (%)	*n* = 16,396 (%)	Odds ratio (95% Cl)	*p*	Adjusted odds ratio (95% Cl)	*p*
Sleeping drugs, n (%)	2047 (12.5)	2643 (16.1)	1.35 (1.27–1.43)	**< 0.001**	1.30 (1.22–1.39)	**< 0.001**
NSAIDs, n (%)	1243 (7.6)	795 (4.8)	0.62 (0.57–0.68)	**< 0.001**	0.61 (0.55–0.67)	**< 0.001**
α-blockers, n (%)	171 (1.0)	414 (2.5)	2.46 (2.1–2.94)	**< 0.001**	2.17 (1.81–2.61)	**< 0.001**
Anti-Parkinson drugs, n (%)	38 (0.2)	30 (0.2)	0.79 (0.49–1.27)	0.33	0.87 (0.53–1.43)	0.59
1st generation H_1_-blockers, n (%)	101 (0.6)	92 (0.6)	0.91 (0.69–1.21)	0.52	0.90 (0.67–1.21)	0.49
H_2_-blockers, n (%)	510 (3.1)	937 (5.7)	1.89 (1.69–2.11)	**< 0.001**	1.79 (1.60-2.00)	**< 0.001**
Drugs for overactive bladder, n (%)	237 (1.4)	408 (2.5)	1.74 (1.48–2.04)	**< 0.001**	1.59 (1.35–1.88)	**< 0.001**
Anti-depressants, n (%)	69 (0.4)	42 (0.3)	0.61 (0.41–0.89)	**< 0.05**	0.54 (0.37–0.80)	**< 0.05**
Anti-emetics, n (%)	95 (0.6)	30 (0.2)	0.32 (0.21–0.47)	**< 0.001**	0.30 (0.19–0.45)	**< 0.001**
Diuretic drugs, n (%)	735 (4.5)	1983 (12.1)	2.93 (2.69–3.20)	**< 0.001**	2.58 (2.36–2.82)	**< 0.001**
Sulpiride, n (%)	112 (0.7)	83 (0.5)	0.74 (0.56–0.98)	**0.038**	0.66 (0.49–0.89)	**< 0.05**
Concomitant intake of multiple antithrombotic drugs, n (%)	155 (0.9)	655 (4.0)	4.36 (3.66–5.20)	**< 0.001**	3.37 (2.81–4.03)	**< 0.001**

Proportions of patients who received each of PIMs categories in diabetic and non-diabetic female groups were analyzed using univariate and multivariate logistic regression analyses. CI, confidence interval

**Table 4:** Sub-group analysis in males: comparisons of prescription rates of each PIMs category after propensity score matching between diabetic and non-diabetic elderly men

Category	Diabetes		Univariate analyses		Multivariate analysis	
	(-)	(+)				
	*n* = 21,210 (%)	*n* = 21,210 (%)	Odds ratio (95% Cl)	*p*	Adjusted odds ratio (95% Cl)	*p*
Sleeping drugs, n (%)	1982 (9.3)	2410 (11.4)	1.24 (1.17–1.32)	**< 0.001**	1.23 (1.15–1.31)	**< 0.001**
NSAIDs, n (%)	1314 (6.2)	623 (2.9)	0.46 (0.42–0.51)	**< 0.001**	0.46 (0.42–0.51)	**< 0.001**
α-blockers, n (%)	203 (1.0)	511 (2.4)	2.55 (2.17–3.01)	**< 0.001**	2.25 (1.91–2.66)	**< 0.001**
Anti-Parkinson drugs, n (%)	49 (0.2)	15 (0.1)	0.31 (0.17–0.55)	**< 0.001**	0.37 (0.20–0.67)	**< 0.05**
1st generation H_1_-blockers, n (%)	134 (0.6)	117 (0.6)	0.87 (0.68–1.12)	0.28	0.87 (0.67–1.13)	0.29
H_2_-blockers, n (%)	691 (3.3)	1159 (5.5)	1.72 (1.56–1.89)	**< 0.001**	1.63 (1.48–1.80)	**< 0.001**
Drugs for overactive bladder, n (%)	277 (1.3)	270 (1.3)	0.97 (0.823–1.15)	0.76	0.91 (0.77–1.08)	0.30
Anti-depressants, n (%)	56 (0.3)	23 (0.1)	0.41 (0.25–0.67)	**< 0.001**	0.40 (0.24–0.67)	**< 0.001**
Anti-emetics, n (%)	97 (0.5)	42 (0.2)	0.43 (0.30–0.62)	**< 0.001**	0.53 (0.36–0.77)	**< 0.001**
Diuretic drugs, n (%)	1080 (5.1)	2356 (11.1)	2.33 (2.16–2.51)	**< 0.001**	2.02 (1.87–2.18)	**< 0.001**
Sulpiride, n (%)	82 (0.4)	28 (0.1)	0.34 (0.22–0.52)	**< 0.001**	0.30 (0.19–0.47)	**< 0.001**
Concomitant intake of multiple antithrombotic drugs, n (%)	594 (2.8)	1717 (8.1)	3.06 (2.78–3.36)	**< 0.001**	2.62 (2.38–2.89)	**< 0.001**

Proportions of patients who received each of PIMs categories in diabetic and non-diabetic male groups were analyzed using univariate and multivariate logistic regression analyses. CI, confidence interval

### Sub-group analyses of prescription rates of PIMs categories in each of older females and males with type 2 diabetes

To focus on older women and men with type 2 diabetes, we conducted the same univariate and multivariate sub-group analyses using the population after excluding ones with type 1 diabetes who were prescribed with insulin only or with insulin in combination with each of two SGLT2 inhibitors, ipragliflozin and dapagliflozin, applicable to type 1 diabetes. The multivariate analysis of prescription rates of PIM categories after propensity score matching in each of older women and men with type 2 diabetes provided essentially similar results (Supplementary Tables S5 and S6) to those obtained from analysis of patients with and without type 1 or type 2 diabetes (see Tables 3 and 4), although the decreased prescription rate of sulpiride was not statistically significant in older women with type 2 diabetes (see Supplementary Table S5).

## Discussion

In the present study, statistical analysis of pharmacy claims data of older individuals who visited one of the nationwide 905 community-based pharmacy branches of Sugi Pharmacy Co., Ltd., after balanced between diabetic and non-diabetic groups for age, gender ratio, and status of home health insurance utilization through PSM, demonstrated significant increases in the total number of medications (except for anti-diabetic drugs), i.e. polypharmacy, and in the incidence of PIMs in older individuals with diabetes, compared to those without diabetes. The present analyses identified five PIM categories regardless of gender and one female-specific PIM category that need special attention in older individuals with diabetes (Table 5). It is to be noted that the identified changes in prescription rates of PIMs categories in each of female and male with diabetes would mainly reflect characteristics of patients with type 2 diabetes, considering the results from the sub-group analysis of female or male patient populations after excluding ones receiving medications for type 1 diabetes (see Supplementary Tables S5 and S6). The evidence obtained in this study would be useful and helpful for pharmacists to check prescriptions for making recommendations for deprescribing of unnecessary or harmful medication in pharmaceutical care of older individuals with diabetes.

**Table 5:** Summary of PIMs that need special attention in the elderly with diabetes

Regardless of genders		Female only
Sleeping drugs; α-blockers; H_2_-blockers; Diuretic drugs; Concomitant intake of multiple antithrombotic drugs		Drugs for overactive bladder

In terms of clinical translation of our findings, the present study provides evidence for PIM categories that need special attention in pharmaceutical care of older patients with diabetes, which should be helpful for pharmacists who are responsible for making recommendations of deprescribing or switching of PIMs. The present evidence, once confirmed by future clinical studies using different data base, might eventually contribute to creation of a diabetes-specific guideline for avoidance of PIMs use in older patients. Actually, we believe that the present findings have some impact on clinical practice. For instance, older individuals with diabetes more often receive benzodiazepine hypnotics that are included in the PIMs list than those without diabetes. In this case, pharmacists may suggest switching to safer medications, such as melatonin receptor agonists and orexin receptor antagonists. The more use of diuretic PIMs in older patients with diabetes could be a risk of dehydration, which should be checked by medical personnel including pharmacists. Concerning the increased prescriptions of anticholinergic PIMs for treatment of overactive bladder in patients with diabetes, pharmacists may consider to make a recommendation for using β_3_ adrenoceptor agonists. Thus, the information obtained in the present study would be useful to promote pharmacist-led optimization of pharmacotherapy.

The increased incidences of three PIM categories, diuretic drugs, sleeping drugs and α-blockers, in individuals with diabetes, identified by statistical analysis of 75,212 older patients (37606 in each of diabetic and non-diabetic groups) after PSM in this study, are essentially in agreement with those detected in older patients with diabetic kidney disease in a recent study analyzing the data of 186 patients [28]. The increased incidence of a PIM category of diuretics in older individuals with diabetes is considered to be associated with the development of diabetic nephropathy [29]. The increased incidence of PIM categories of diuretics, α-blockers and concomitant intake of multiple antithrombotic drugs in older individuals with diabetes might be associated with the development of cardiovascular diseases including hypertension, coronary heart disease, cerebrovascular disease and heart failure, in which diabetes is a major risk factor [30, 31]. There is evidence for the link between anxiety or sleep disorders and diabetes [32, 33] and for the association between gastrointestinal symptoms and insomnia in older individuals with diabetes [34]. These reports might interpret the increased incidence of PIM categories of sleeping drugs and H_2_-blockers in older individuals with diabetes. The possibility cannot be ruled out that the high incidence of the PIM category of H_2_-blockers may be associated with the gastric damage following the polypharmacy that is more common in older individuals with diabetes than those without diabetes (see Fig. 1).

Of interest was that the increased incidence of a PIM category, drugs for overactive bladder, in the diabetic group was detected in older adult women, but not men, by sub-group analysis in each gender (aORs were 1.59 and 0.91, respectively) (see Tables 3, 4 and 5). Drugs for overactive bladder included in the PIMs list are exclusively anti-cholinergic/anti-muscarinic agents (see Supplementary materials; Table S1), which are associated an increased risk of dementia in older individuals [35, 36]. Therefore, pharmacists may consider the possibility to make recommendations of deprescribing anti-cholinergic agents and prescribing alternative medication, such as β_3_-adrenoceptor agonists, particularly in older women with diabetes. It is still open to question why the diabetes-related increase in the incidence of drugs for overactive bladder included in the PIMs list was detected only in females. In general, the prevalence of overactive bladder increases with menopausal stage in women [37]. Therefore, older women with diabetes enrolled in this study are considered to have a postmenopause-related risk for overactive bladder. The increased use of diuretic drugs in older individuals with diabetes regardless of gender, as mentioned above, could be associated with the symptoms of overactive bladder, particularly in older women with diabetes who have the postmenopausal risk for overactive bladder, which might contribute to the increased incidence of the PIM category of drugs for overactive bladder. There is evidence that age-related increase in body mass index (BMI) in women is associated with the development of overactive bladder [38], which is consistent with the high risk for overactive bladder in older women with diabetes in the present study, because the majority of the diabetic group are considered older women with type 2 diabetes that is related to obesity.

PIM categories that showed lower incidence in older individuals with diabetes were NSAIDs, anti-Parkinson drugs, anti-emetics, and sulpiride (see Table 2). Although we have no evidence, it is speculated that physicians might hesitate in prescribing NSAIDs, known to reduce renal function, and sulpiride, known to cause high insulin and high glucose [39], for patients with diabetes. The reasons for lower incidence of prescriptions of anti-Parkinson drugs and anti-emetics remain unclear.

There are some limitations of the present study. Pharmacy claims data include only limited information of patients, so that it is not possible to eliminate bias due to unknown confounding factors other than the age, gender and status of home healthcare insurance applications. In particular, the lack of information about common chronic conditions, which should be ideally included as confounding factors in matching, is one of weak points in the present study. Another problem in this study is that pharmacy claims data in Japan do not include disease name information, so that the identification of individuals with diabetes had to be done by estimation on the basis of anti-diabetic medications. The classification of patients solely according to prescriptions may cause potential misclassification, particularly of older patients with diabetes who were managed by controlling diet or lifestyle without diabetes medications. Furthermore, this study did not analyze whether the types or number of anti-diabetic medications would differently affect PIM characteristics of older individuals with diabetes. Additionally, we could not capture the clinical intent behind prescriptions, e.g., physician’s deliberate prescribing of PIMs for symptom control, through pharmacy claims data.

## Conclusion

In the present study to characterize diabetes-related risky PIMs in older individuals using pharmacy claims data, we identified five PIM categories, sleeping drugs, α-blockers, H_2_-blockers, diuretic drugs, and concomitant intake of multiple antithrombotic drugs that need special attention in the older individuals with diabetes regardless gender, and detected drugs for overactive bladder as a diabetes-related high incidence PIM category only for females. The gender-specific increase in the use of PIMs for treatment of overactive bladder is particularly interesting, but needs to be confirmed by statistical analysis of different database including more information of each patient after multiple comparison adjustments in future. To establish the diabetes-specific PIM characterization, as suggested by the present study, prospective approaches, such as interventional studies targeting pharmacist-led deprescribing of PIMs in older patients with diabetes, might be necessary. Nonetheless, the present study provides useful information in pharmacy practice, which might contribute to creation of deprescribing checklists, interdisciplinary care strategies or educational intervention programs for pharmaceutical care of older patients with diabetes. Together, we believe that detailed characterization of disease-specific high risk PIM categories in older individuals would be beneficial for pharmacists who participate in pharmaceutical care for geriatric patients with different disease backgrounds.

## Supplementary Information

Below is the link to the electronic supplementary material.

Supplementary Material 1

## Data Availability

The data supporting the findings in the present study are available from the corresponding author upon reasonable request.

## Abbreviations

aORs: Adjusted odds ratios
BMI: Body mass index
CI: Confidence intervals
ORs: Odds ratios
PIM: Potentially inappropriate medication
PSM: Propensity score matching
VIF: Variance inflation factor
