# Supplementary material for: Identification of potentially inappropriate medications characteristic of older individuals with diabetes: a study using pharmacy claims data
Source: J Pharm Health Care Sci. 2025 Nov 12;11:100. doi: 10.1186/s40780-025-00505-7 (PMC12613534; doi:10.1186/s40780-025-00505-7)
Supplement: Supplementary file 1 — Supplementary Material 1 [file 40780_2025_505_MOESM1_ESM.pdf]

Table S1 A list of PIMs (except for anti-diabetic drugs) released by the Japanese Geriatrics Society as 'Medications that Require Particularly Careful Administration in the Guidelines for the Safety of Pharmacotherapy in the Elderly in 2015'

Sleeping drugs<br>Benzodiazepines: Alprazolam, Bromazepam, Brotizolam, Chlordiazepoxide, Clorazepate dipotassium, Clotiazepam, Cloxazolam, Diazepam, Estazolam, Ethyl loflazepate, Etizolam, Fludiazepam, Flunitrazepam, Flurazepam hydrochloride, Flutazolam, Flutoprazepam, Haloxazolam, Lorazepam, Lormetazepam, Medazepam, Mexazolam, Nimetazepam, Nitrazepam, Oxazolam, Quazepam, Rilmazafone hydrochloride hydrate, Tofisopam, Triazolam<br>Non-Benzodiazepines (Z-drugs): Eszopiclone, Zolpidem tartrate, Zopiclone
Anti-depressants<br>Amitriptyline hydrochloride
Sulpiride<br>Sulpiride
Anti-Parkinson drugs<br>Anticholinergic drugs: Biperiden hydrochloride, Mazaticol hydrochloride hydrate, Piroheptine hydrochloride, Profenamine hibenzate, Profenamine hydrochloride, Promethazine hibenzate, Promethazine hydrochloride, Promethazine methylenedisalicylate, Trihexyphenidyl hydrochloride
Concomitant intake of multiple antithrombotic drugs (antiplatelet agents and/or anticoagulants)<br>Aspirin, Apixaban, Cilostazol, Clopidogrel sulfate, Dabigatran etexilate methanesulfonate, Edoxaban tosilate hydrate, Prasugrel hydrochloride, Rivaroxaban, Ticagrelor, Ticlopidine hydrochloride, Warfarin potassium
Diuretic drugs<br>Loop diuretics: Azosemide, Bumetanide, Furosemide, Piretanide, Torasemide,<br>Aldosterone antagonists: Eplerenone, Spironolactone
α-adrenoceptor blockers<br>Bunazosin hydrochloride, Doxazosin mesylate, Prazosin hydrochloride, Terazosin hydrochloride hydrate, Urapidil

H_1-blockers (1st generation) including their mixture<br>Alimemazine tartrate, Clemastine fumarate, Cyproheptadine hydrochloride hydrate, d-Chlorpheniramine maleate, Diphenhydramine hydrochloride, Homochlorcyclizine hydrochloride, Hydroxyzine hydrochloride, Hydroxyzine pamoate, Promethazine hibenzate, Promethazine hydrochloride, Promethazine methylenedisalicylate
H_2-blockers<br>Cimetidine, Famotidine, Lafutidine, Nizatidine, Ranitidine hydrochloride, Roxatidine acetate hydrochloride
Antiemetics<br>Metoclopramide, Prochlorperazine maleate, Promethazine hibenzate, Promethazine hydrochloride, Promethazine methylenedisalicylate
Drugs for overactive bladder<br>Muscarinic receptor antagonist; Oxybutynin hydrochloride, Fesoterodine fumarate, Imidafenacin, Propiverine hydrochloride, Solifenacin succinate, Tolterodine tartrate
Non-steroidal anti-inflammatory drugs (NSAIDs)<br>Acemetacin, Amfenac sodium, Ampiroxicam, Bucolome, Diclofenac sodium, Emorfazone, Etodolac, Ibuprofen, Indometacin, Indometacin farnesil, Nabumetone, Naproxen, Oxaprozin, Piroxicam, Pranoprofen, Sulindac, Tiaprofenic acid, Tiaramide hydrochloride, Zaltoprofen

PIMs designated only for patients with specific diseases were not included in this list.

Table S2 List of prescribed anti-diabetic agents for identification of diabetic patients

Class name	Generic name
Insulin	Aspart
	Degludec
	Degludec / Aspart
	Glargine
	Glulisine
	Lispro
Glucagon-like peptide-1 receptor agonist	Dulaglutide
	Exenatide
	Liraglutide
	Lixisenatide
Sulfonylurea	Acetohexamide
	Chlorpropamide
	Glibenclamide
	Gliclazide
	Glimepiride
	Glyclopyramide
	Tolbutamide
Glinide	Mitiglinide calcium hydrate
	Nateglinide
	Repaglinide
Biguanide	Buformin hydrochloride
	Metformin hydrochloride
Alpha-glucosidase inhibitor	Acarbose
	Miglitol
	Voglibose
DPP-4 inhibitor	Alogliptin benzoate
	Anagliptin
	Linagliptin
	Omarigliptin
	Saxagliptin hydrate
	Sitagliptin phosphate hydrate
	Teneligliptin hydrobromide hydrate
	Trelagliptin succinate
	Vildagliptin
Sodium-glucosecotransporter-2 inhibitor	Canagliflozin hydrate
	Dapagliflozin propylene glycolate hydrate

	Empagliflozin<br>Ipragliflozin L-proline<br>Luseogliflozin hydrate<br>Tofogliflozin hydrate
Insulin sensitizer	Pioglitazone hydrochloride
Drug combination	Alogliptin benzoate / Metformin hydrochloride<br>Alogliptin benzoate / Pioglitazone hydrochloride<br>Anagliptin / Metformin hydrochloride<br>Empagliflozin / Linagliptin<br>Mitiglinide calcium hydrate / Voglibose<br>Pioglitazone hydrochloride / glimepiride<br>Pioglitazone hydrochloride / Metformin hydrochloride<br>Sitagliptin phosphate hydrate / Ipragliflozin L-proline<br>Teneligliptin hydrobromide hydrate / Canagliflozin hydrate<br>Vildagliptin / Metformin hydrochloride

Table S3 Patients' characteristics in diabetic and non-diabetic groups of the female elderly before and after propensity score matching

	Before propensity score matching			After propensity score matching		
	Diabetes			Diabetes		
	(-)	(+)	*p*	(-)	(+)	*p*
Characteristics	n=171911	n=16396		n=16396	n=16396	
Age, Median [range]	76 [65-107]	75 [65-103]	**< 0.001**	75 [65-103]	75 [65-103]	1.00
Use of home healthcare insurance, n (%)	12004 (7.0)	1305 (8.0)	**< 0.001**	1305 (8.0)	1305 (8.0)	1.00

Statistical comparisons of age between diabetes and non- diabetes groups were performed using Mann-Whitney's *U* test, and the differences in the status of home healthcare insurance application were analyzed by Fisher's exact test.

Table S4 Patients' characteristics in diabetic and non-diabetic groups of the male elderly before and after propensity score matching.

	Before propensity score matching			After propensity score matching		
	Diabetes			Diabetes		
	(-)	(+)	*p*	(-)	(+)	*p*
Characteristics	n=124352	n=21210		n =21210	n=21210	
Age, Median [range]	75 [65-104]	73 [65-107]	**< 0.001**	73 [65-104]	73 [65-107]	1.00
Use of home healthcare insurance, n (%)	5227 (4.2)	836 (3.9)	0.081	821 (3.9)	836 (3.9)	0.73

Statistical comparisons of age between diabetes and non- diabetes groups were performed using Mann-Whitney's *U* test, and the differences in the status of home healthcare insurance application were analyzed by Fisher's exact test.

Table S5 Sub-group analysis of prescription rates of each PIMs category after propensity score matching in older females with and without type 2 diabetes

Category	diabetes (-) n= 16396 (%)	diabetes (+) n=13956 (%)	Univariate analyses odds ratio (95% CI)	Univariate analyses *p*	Multivariate analysis Adjusted odds ratio (95% CI)	Multivariate analysis *p*
Sleeping drugs, n (%)	2047 (12.5)	2289 (16.4)	1.38 (1.29-1.47)	**< 0.001**	1.33 (1.24-1.42)	**< 0.001**
NSAIDs, n (%)	1243 (7.6)	685 (4.9)	0.63 (0.57-0.69)	**< 0.001**	0.61 (0.56-0.68)	**< 0.001**
α-blockers, n (%)	171 (1)	334 (2.4)	2.33 (1.93-2.80)	**< 0.001**	2.08 (1.72-2.51)	**< 0.001**
Anti-Parkinson drugs, n (%)	38 (0.2)	30 (0.2)	0.93 (0.57-1.50)	0.76	1.01 (0.62-1.65)	0.97
1st generation H1-blockers, n (%)	101 (0.6)	84 (0.6)	0.98 (0.73-1.31)	0.88	0.95 (0.71-1.29)	0.75
H2-blockers, n (%)	510 (3.1)	803 (5.8)	1.90 (1.70-2.13)	**< 0.001**	1.81 (1.61-2.03)	**< 0.001**
Drugs for overactive bladder, n (%)	237 (1.4)	371 (2.7)	1.86 (1.58-2.20)	**< 0.001**	1.70 (1.44-2.02)	**< 0.001**
Anti-depressants, n (%)	69 (0.4)	33 (0.2)	0.56 (0.37-0.85)	**< 0.05**	0.49 (0.32-0.75)	**< 0.05**
Anti-emetics, n (%)	95 (0.6)	24 (0.2)	0.30 (0.19-0.46)	**< 0.001**	0.28 (0.17-0.44)	**< 0.001**
Diuretic drugs, n (%)	735 (4.5)	1658 (11.9)	2.87 (2.63-3.14)	**< 0.001**	2.54 (2.32-2.78)	**< 0.001**
Sulpiride, n (%)	112 (0.7)	81 (0.6)	0.85 (0.64-1.13)	0.26	0.75 (0.56-1.00)	0.053
Concomitant intake of multiple antithrombotic drugs, n (%)	155 (0.9)	528 (3.8)	4.12 (3.44-4.94)	**< 0.001**	3.18 (2.65-3.83)	**< 0.001**

To extract patients with type 2 diabetes, we excluded older women who received insulin only or insulin in combination with each of two SGLT2 inhibitors, ipragliflozin and dapagliflozin, applicable to type 1 diabetes, and balanced the enrolled patients with and without type 2 diabetes by propensity score matching. Proportions of patients who received each of PIMs categories in the two groups with and without type 2 diabetes were analyzed using univariate and multivariate logistic regression analyses. CI, confidence interval.

Table S6 Sub-group analysis of prescription rates of each PIMs category after propensity score matching in older males with and without type 2 diabetes

Category	diabetes (-) n= 21210 (%)	diabetes (+) n=18185 (%)	Univariate analyses odds ratio (95% Cl)	Univariate analyses *p*	Multivariate analysis Adjusted odds ratio (95% Cl)	Multivariate analysis *p*
Sleeping drugs, n (%)	1982 (9.3)	2069 (11.4)	1.25 (1.17-1.33)	**< 0.001**	1.23 (1.15-1.32)	**< 0.001**
NSAIDs, n (%)	1314 (6.2)	555 (3.1)	0.48 (0.43-0.53)	**< 0.001**	0.49 (0.44-0.54)	**< 0.001**
α-blockers, n (%)	203 (1)	430 (2.4)	2.51 (2.12-2.96)	**< 0.001**	2.20 (1.85-2.61)	**< 0.001**
Anti-Parkinson drugs, n (%)	49 (0.2)	13 (0.1)	0.31 (0.17-0.57)	**< 0.001**	0.38 (0.20-0.71)	**< 0.05**
1st generation H1-blockers, n (%)	134 (0.6)	107 (0.6)	0.93 (0.72-1.20)	0.58	0.93 (0.71-1.21)	0.58
H2-blockers, n (%)	691 (3.3)	995 (5.5)	1.72 (1.56-1.90)	**< 0.001**	1.63 (1.47-1.80)	**< 0.001**
Drugs for overactive bladder, n (%)	277 (1.3)	238 (1.3)	1.00 (0.84-1.19)	0.98	0.95 (0.79-1.13)	0.54
Anti-depressants, n (%)	56 (0.3)	21 (0.1)	0.44 (0.26-0.72)	**< 0.05**	0.44 (0.26-0.74)	**< 0.05**
Anti-emetics, n (%)	97 (0.5)	38 (0.2)	0.46 (0.31-0.66)	**< 0.001**	0.55 (0.38-0.81)	**< 0.05**
Diuretic drugs, n (%)	1080 (5.1)	1993 (11)	2.29 (2.12-2.48)	**< 0.001**	1.98 (1.83-2.14)	**< 0.001**
Sulpiride, n (%)	82 (0.4)	26 (0.1)	0.37 (0.24-0.57)	**< 0.001**	0.32 (0.21-0.51)	**< 0.001**
Concomitant intake of multiple antithrombotic drugs, n (%)	594 (2.8)	1492 (8.2)	3.10 (2.81-3.42)	**< 0.001**	2.66 (2.41-2.94)	**< 0.001**

To extract patients with type 2 diabetes, we excluded older men who received insulin only or insulin in combination with each of two SGLT2 inhibitors, ipragliflozin and dapagliflozin, applicable to type 1 diabetes, and balanced the enrolled patients with and without type 2 diabetes by propensity score matching. Proportions of patients who received each of PIMs categories in the two groups with and without type 2 diabetes were analyzed using univariate and multivariate logistic regression analyses. CI, confidence interval.

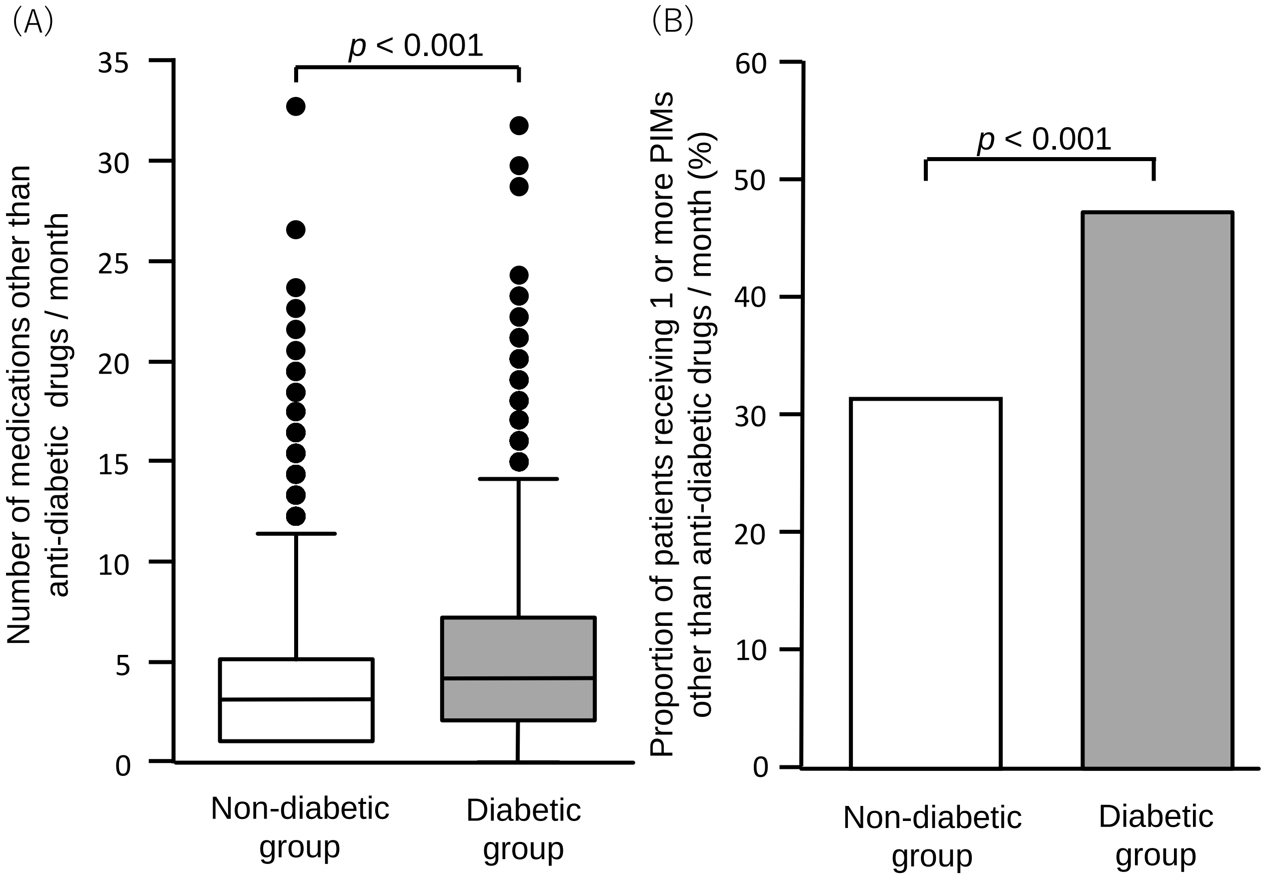

Supplementary Figure S1. Polypharmacy and increased incidence of PIMs in older patients with diabetes, when evaluated by analysis of prescriptions only in the first visit during the one-month observation period. (A) The number of medications other than anti-diabetic drugs prescribed over a period of 1 month in diabetic and non-diabetic groups after propensity score matching. Data show the median with upper and lower quartiles, as well as the maximum and minimum values, while outliers are indicated as dots. (B) Proportion of patients receiving PIMs other than anti-diabetic drugs over a period of 1 month in diabetic and non-diabetic groups. Statistical analysis of the differences between diabetic and non-diabetic groups was performed using Mann-Whitney's *U* test (A) or Fisher's exact test (B).
